# Analysis of the current status and associated factors of innovative leadership among male nurses in China: a cross-sectional study

**DOI:** 10.3389/fmed.2026.1920462

**Published:** 2026-10-14

**Authors:** Caixia Ma, Xin Chen, Zhen Li, Peiyao Zhu, Mengge Zhang, Jingwen Cao, Jie Kou, Hengyu Hu

**Affiliations:** 1 Pediatric Department, Henan Provincial People's Hospital, Zhengzhou, Henan, China; 2 School of Nursing, Henan Medical University, Xinxiang, Henan, China; 3 Breast Surgery, Henan Provincial People's Hospital, Zhengzhou, Henan, China; 4 School of Nursing and Health, Henan University, Kaifeng, Henan, China; 5 School of Nursing, Zhengzhou University, Zhengzhou, Henan, China; 6 Neurological ICU, Henan Provincial People's Hospital, Zhengzhou, Henan, China; 7 Nursing Department, Henan Provincial People's Hospital, Zhengzhou, Henan, China

**Keywords:** a cross-sectional study, associated factors, critical thinking, innovative leadership, male nurse

## Abstract

**Objective:**

To investigate the current state of innovative leadership among male nurses in Grade A, Level 3 hospitals in Henan Province, China, and analyze factors associated with innovative leadership, thereby providing an empirical basis for developing targeted intervention strategies.

**Methods:**

Using convenience sampling, 222 male nurses from eight Grade A, Level 3 hospitals in Henan Province, China, were selected as study participants between February and June 2025. The survey was conducted using a general data questionnaire, the Innovative Leadership Scale, and the Clinical Thinking Diagnostic of Frontline Nurses.

**Results:**

The median score of innovative leadership among male nurses was 154.00 (141.00, 168.00). Innovative leadership was positively correlated with critical thinking (*r* = 0.663, *P* < 0.01). In an enter multiple linear regression model, job role, number of published papers, participation in innovative leadership conferences/training, and critical thinking were independently associated with innovative leadership (*P* < 0.05), and the model explained 76.4% of the total variance.

**Conclusion:**

Male nurses working in tertiary hospitals in Henan Province showed a moderate level of innovative leadership. Nursing managers should monitor male nurses' innovative thinking and practical behaviors, and implement personalized strategies based on associated factors to support the development of innovative leadership and continuous improvement in nursing service quality.

## 1 Introduction

Healthcare systems around the world are facing multiple challenges, including the growing burden of chronic disease care, frequent public health emergencies, and comprehensive innovations in medical technology. The traditional nursing service model centered on executing physicians’ orders can no longer satisfy the requirements for high-quality modern nursing development. Nursing staff are in urgent need of profound role transformation toward active decision-making and innovative clinical practice (1). The World Health Organization has clearly stated that cultivating nursing leadership serves as a core strategy to realize universal health coverage and Sustainable Development Goals. Specifically, innovative leadership plays an indispensable role in boosting the high-quality development of global nursing services (2). Understanding how healthcare professionals conceptualize leadership traits is essential for developing effective leadership development programs. Recent qualitative research has identified that healthcare managers value traits such as strategic thinking, communication competence, and change orientation in their implicit leadership theories, which aligns with the dimensions of innovative leadership measured in our study (3).

While general leadership emphasizes coordinating team efforts and maintaining efficient operations to achieve organizational goals, innovative leadership builds upon this foundation by placing greater weight on proactively identifying opportunities for change, tolerating constructive failure, and driving systemic transformation. Its core connotation involves actively proposing, testing and standardizing novel concepts, technologies and workflows, as well as facilitating sustained optimization and organizational innovation through building an open, inclusive and supportive workplace atmosphere (4). For nursing practitioners, innovative leadership embodies the capacity to resolve practical clinical dilemmas, proactively detect workplace deficiencies, and leverage scientific thinking to optimize nursing service frameworks, upgrade nursing techniques and reconstruct doctor-nurse collaboration mechanisms. Meanwhile, it encompasses integrated capabilities to unite nursing teams for joint innovation and construct an inclusive atmosphere that encourages trial and error. Existing evidence indicates that innovative leadership can effectively stimulate staff innovative performance, cultivate team innovation culture, elevate job satisfaction, lower turnover rates and facilitate long-term institutional growth (5). At the organizational level, healthcare system reforms and efficiency improvements are contingent upon the development of leadership competencies among healthcare professionals. A systematic review of hospital efficiency in developing countries demonstrated that systemic interventions can significantly improve organizational performance metrics, suggesting that investments in leadership development may have cascading benefits for healthcare quality and efficiency (6).

Based on higher-order thinking skill theory, individual cognitive capacities including critical thinking, problem solving, decision-making and meta-cognition act as core motivators of innovative behaviors and innovative leadership practice (7). Critical thinking stands out as the most vital component among these abilities. It refers to people's capability to flexibly apply accumulated knowledge and experience in complex scenarios to infer, analyze and assess clinical evidence, so as to conduct self-correction and formulate scientific, reasonable clinical decisions (8). Nurses equipped with advanced critical thinking skills can identify the root causes of complicated clinical problems and break rigid inherent thinking modes to create innovative solutions. Such nurses constitute the inherent core power to motivate peers to carry out innovative nursing practices and exert personal leadership (9, 10).

Although the value of innovative leadership has attracted increasing attention, empirical research on innovative leadership at the individual nurse level remains limited, and in-depth studies on specific subgroups within the nursing workforce are particularly scarce (11). The proportion of male nurses has risen gradually in recent years, yet male nurses still account for a relatively low percentage of nursing personnel in many countries (12, 13). As a gender minority in this traditionally female dominated profession, male nurses may encounter unique social expectations, occupational stereotypes, organizational management patterns and career development experiences (14, 15). These contextual factors may shape their opportunities to take part in decision-making, quality improvement work and leadership development (16). Nevertheless, existing studies on male nurses mostly focus on occupational adaptation, professional identity, gender related experiences and career progression (17, 18), while research on nursing leadership generally treats nurses as an entire group. Accordingly, adequate baseline evidence is still lacking concerning the innovative leadership level of male nurses and its relevant individual and organizational factors. Importantly, the purpose of this study is not to test gender related mechanisms directly, but to establish baseline evidence in this under-represented subgroup. Research on this under-represented subgroup enables a more detailed comprehension of innovative leadership issues inside the gender diversified nursing workforce and supplies evidence for developing suitable and targeted leadership support strategies.

In conclusion, this cross-sectional study targets male nurses working in tertiary hospitals in Henan Province, China, as a unique subgroup to explore their current innovative leadership status and relevant associated factors. The results can offer evidence for nursing managers to formulate targeted training plans, optimize clinical nursing human resource management systems, support male nurses' innovative potential, promote diversified construction of nursing teams, and continuously improve the overall quality of clinical nursing services.

## 2 Materials and methods

### 2.1 Setting and participants

Using convenience sampling, male nurses from eight Grade A, Level 3 hospitals in Henan Province, China, were selected as study participants between February and June 2025. Inclusion criteria: (1) Hold a registered nurse license and have ≥1 year of clinical experience; (2) In good physical and mental health, with no history of mental or psychological disorders; (3) Have provided informed consent and voluntarily participate in this study. Exclusion criteria: (1) Nurses on advanced training, nurses in standardized training programs, nursing assistants, and nursing interns; (2) Those unable to complete the questionnaire due to sick leave or off-site training.

*A priori* sample size estimation was performed using G*Power 3.1.9 the primary outcome was continuous and was planned to be analyzed using multiple linear regression. With a significance level of *α* = 0.05, power (1−*β*) = 0.95, an expected medium effect size of *f*^2^ = 0.15, and a conservative assumption of up to 10 predictors in the regression model, the minimum required total sample size was calculated as 172. Considering potentially invalid or incomplete electronic questionnaires, and assuming an invalid response rate of approximately 20%, at least 172/0.8 ≈ 215 participants were required. In the formal survey, 240 questionnaires were collected; after data cleaning, 222 valid questionnaires remained.

The study adhered to the principles of the Declaration of Helsinki and received approval from the Ethics Committee of Henan Provincial People's Hospital [Ethics Approval No. (2024) Lun Shen No. (114)].

### 2.2 Measurement

#### 2.2.1 General information questionnaire

These variables were designed by the researchers based on a review of relevant literature and in consultation with experts and include age, educational level, marital status, professional title, department, average frequency of night shifts, job role, employment status, number of published papers, and attendance at an innovation leadership conference/training session.

#### 2.2.2 Innovative leadership scale (ILS)

This scale was developed by Kemer et al. (19) and adapted into Chinese by our research team (20). It comprises five dimensions—being a changer, motivating others, being open to new ideas, being a leader, and being energetic—with a total of 43 items. It employs a 5-point Likert scale ranging from 1 (strongly disagree) to 5 (strongly agree), with 17 items reverse-scored. The total score ranges from 43 to 215, with higher scores indicating a higher level of innovative leadership. In this study, the Cronbach's *α* coefficient for this scale was 0.911.

#### 2.2.3 Critical thinking diagnostic of frontline nurses (CTDfn)

This scale was developed by Berkow et al. (21) and adapted into Chinese by Zhang et al. (22). It comprises five dimensions—problem recognition, clinical decision making, prioritization, nursing planning, and nursing plan implementation and improvement—and consists of 16 items in total. It uses a 6-point Likert scale ranging from 1 (strongly disagree) to 6 (strongly agree), with a total score ranging from 16 to 96; a higher score indicates a higher level of critical thinking among nurses. In this study, the Cronbach's *α* coefficient for this scale was 0.941.

### 2.3 Data collection

Our study collected data through an electronic questionnaire administered via the Questionnaire Star platform (https://www.wjx.cn/). After obtaining approval from the nursing departments of each hospital, links to the electronic questionnaire were distributed via DingTalk groups. A standardized instruction explained the purpose, significance, and completion procedures of the survey, and participants completed the questionnaire independently and anonymously based on informed consent. Each IP address was limited to one response.

To ensure data quality, we established the exclusion threshold based on response time distributions from a pilot sample (*n* = 35) drawn from the same target population. The pilot data showed a median completion time of 312 s and a 5th percentile of 156 s. Drawing on established web-survey quality control practices, we adopted a relative threshold approach and set the cutoff at 180 s—which is more conservative than the pilot 5th percentile and corresponds to approximately 0.58 times the pilot median. A total of 240 raw questionnaires were gathered in the formal investigation. During data cleaning, 11 responses were eliminated for completion time shorter than 180 s, and another 7 samples were excluded owing to repetitive answer patterns (operationally defined as >80% identical consecutive choices, following the longstring index). After the two-step screening procedure, 222 valid questionnaires remained, yielding a questionnaire retention rate of 92.5%. This time-based criterion was used to identify responses with a high likelihood of insufficient item engagement, rather than as a definitive indicator of individual respondent diligence.

### 2.4 Statistical analysis

Statistical analyses were performed with SPSS 26.0. Categorical variables are presented as frequencies and percentages. Given the non-normal distribution of continuous scale data, median (*M*) and the 25th and 75th percentiles (*P*_25_, *P*_75_) were used for descriptive characterization. Non-parametric tests were applied for univariate comparisons. Spearman rank correlation was used to examine correlations. For multivariable analysis, ordinary least-squares (OLS) multiple linear regression was performed using the enter method. Regression assumptions were examined using the variance inflation factor (VIF) for multicollinearity, partial regression plots and residual scatterplots for linearity, residual-vs.-predicted plots for homoscedasticity, Q-Q plots, residual histograms, and the Shapiro–Wilk test for normality, and the Durbin–Watson statistic for independence. Influential observations were assessed using standardized residuals, leverage values, and Cook's distance. The significance level was set at *α* = 0.05. Prior to analysis, 17 negatively worded items from the ILS were reverse recoded in SPSS using the formula “new value =  6 − original value”. Recoding accuracy was verified by checking frequency distributions and recalculating Cronbach's *α* (*α* = 0.911).

## 3 Results

### 3.1 Common method bias test

Before data analysis, Harman's single-factor test was performed to assess potential common method bias (CMB) (23). Twenty-three factors had eigenvalues greater than 1, and the first factor accounted for 28.07% of the variance, which is below the recommended 40% threshold. Based on this specific diagnostic, the findings did not suggest substantial common-method variance. However, because all principal variables were collected using self-report measures in a single survey, common-method bias cannot be completely excluded.

### 3.2 Scores on ILS and CTDfn among male nurses

This study included a total of 222 male nurses, whose ILS scores averaged 154.00 (141.00, 168.00), with an average score of 3.58 (3.28, 3.91) per item. In addition, their CTDfn scores averaged 76.00 (69.00, 82.00).

### 3.3 A comparison of innovative leadership scores among male nurses with different characteristics

Of the 222 male nurses, the majority were under 40 years of age (93.7%), held a bachelor's degree (88.3%), and were married (55.9%). Univariate analysis revealed that the average number of night shifts (per month), job role, employment status, number of published papers, and attending an innovation leadership conference/training session were associated with male nurses' innovation leadership (*P* < 0.05) (see Table 1).

**Table 1:** Univariate analysis of innovative leadership scores among male nurses with different characteristics (*N* = 222).

Variables	Groups	*N* (%)	*M* (*P*_25_, *P*_75_)	*Z*/*H*	*P*
Age (years)	<30	118 (53.2)	152.00 (146.00, 164.00)	5.734^a^	0.057
	31–40	90 (40.5)	157.00 (138.00, 169.25)		
	>40	14 (6.3)	176.00 (142.00, 183.00)		
Educational level	Associate's degree	2 (0.9)	142.00 (142.00, 142.00)	2.243^a^	0.326
	Bachelor's degree	196 (88.3)	154.00 (141.00, 168.00)		
	Master's degree or higher	24 (10.8)	163.00 (140.25, 177.25)		
Marital status	Unmarried	98 (44.1)	152.00 (143.75, 161.50)	−1.482^b^	0.138
	Married	124 (55.9)	157.00 (140.00, 170.00)		
Professional title level	Nurse	66 (29.7)	154.00 (146.75, 164.25)	6.774^a^	0.079
	Senior nurse	64 (28.8)	150.50 (141.00, 157.75)		
	Nurse-in-charge	84 (37.9)	162.50 (139.00, 172.00)		
	Associate chief nurse and above	8 (3.6)	165.00 (140.50, 178.25)		
Department	Emergency department	40 (18.0)	156.00 (137.50, 164.00)	1.162^a^	0.884
	Intensive care unit	87 (39.2)	153.00 (144.00, 166.00)		
	Operating room	48 (21.6)	155.50 (141.00, 168.00)		
	Hemodialysis department	15 (6.8)	154.00 (143.00, 169.00)		
	Other	32 (14.4)	159.00 (135.25, 175.25)		
Average number of night shifts (per month)	≤1	54 (24.3)	163.00 (152.00, 171.00)	16.675^a^	**0.001**
	2–4	44 (19.8)	157.00 (139.00, 172.00)		
	5–7	64 (28.9)	152.50 (141.50, 159.75)		
	≥8	60 (27.0)	148.00 (137.00, 164.00)		
Job role	Nurse	164 (73.9)	150.50 (139.00, 163.00)	41.445^a^	**<0.001**
	Team leader	34 (15.3)	159.00 (149.25, 175.50)		
	Head nurse	24 (10.8)	174.50 (163.75, 186.50)		
Employment status	Permanent staff	14 (6.3)	164.00 (158.00, 182.00)	8.062^a^	**0.018**
	Human resources services	134 (60.4)	156.00 (140.00, 169.00)		
	Contract employment	74 (33.3)	152.00 (144.75, 160.25)		
Average monthly income (CNY)	<5,000	24 (10.8)	148.50 (143.75, 162.50)	0.679^a^	0.712
	5,000–10,000	114 (51.4)	154.00 (143.75, 168.00)		
	>10,000	84 (37.8)	156.00 (138.00, 173.00)		
Specialty nurse	No	110 (49.5)	157.00 (139.00, 170.00)	−0.920^b^	0.358
	Yes	112 (50.5)	152.00 (144.25, 166.75)		
Number of published papers	0	149 (67.1)	147.00 (138.00, 154.50)	151.534^a^	**<0.001**
	1–2	44 (19.8)	168.00 (166.00, 170.75)		
	3–4	19 (8.6)	182.00 (176.00, 185.00)		
	≥5	10 (4.5)	189.00 (186.50, 191.75)		
Attend an innovation leadership conference/training session	No	168 (75.7)	149.50 (139.00, 157.75)	−11.022^b^	**<0.001**
	Yes	54 (24.3)	176.00 (170.00, 185.00)		

Bolded numbers indicate *P* < 0.05.

*P* < 0.05 indicates statistically significant difference between groups.

a Kruskal–Wallis *H* test.

b Mann–Whitney *U Z* test.

### 3.4 Analysis of the correlation between innovative leadership and critical thinking among male nurses

The results of the Spearman correlation analysis show that male nurses' innovative leadership is positively correlated with critical thinking (*r* = 0.663, *P* < 0.01), and there are also positive correlations among the various dimensions, as detailed in Table 2.

**Table 2:** Analysis of the correlation between innovative leadership and critical thinking among male nurses.

Variables	1	2	3	4	5	6	7	8	9	10	11	12
1	–											
2	0.658**	–										
3	0.696**	0.192**	–									
4	0.760**	0.590**	0.361**	–								
5	0.801**	0.524**	0.381**	0.665**	–							
6	0.679**	0.182**	0.694**	0.323**	0.353**	–						
7	0.663**	0.269**	0.673**	0.439**	0.462**	0.727**	–					
8	0.551**	0.260**	0.562**	0.343**	0.379**	0.630**	0.867**	–				
9	0.568**	0.217**	0.601**	0.375**	0.376**	0.621**	0.845**	0.678**	–			
10	0.637**	0.292**	0.526**	0.439**	0.521**	0.632**	0.876**	0.733**	0.682**	–		
11	0.558**	0.159*	0.595**	0.409**	0.389**	0.634**	0.893**	0.667**	0.691**	0.742**	–	
12	0.500**	0.176**	0.593**	0.306**	0.314**	0.602**	0.848**	0.668**	0.651**	0.650**	0.812**	–

1, Innovation leadership scale; 2, changer; 3, motivating others; 4, energetic; 5, being open-minded; 6, being a leader; 7, critical thinking diagnostic of frontline nurses; 8, problem recognition; 9, clinical decision making; 10, prioritization; 11, nursing planning; 12, nursing implementation and improvement.

* *P* < 0.05.

** *P* < 0.01.

### 3.5 Multivariable analysis of factors associated with innovative leadership among male nurses

Candidate variables were selected *a priori* based on previous literature and clinical relevance and were entered simultaneously into an enter multiple linear regression model, with the total ILS score as the dependent variable. The assignment of categorical independent variables is shown in Table 3. The model was statistically significant (*R*^2^ = 0.777, adjusted *R*^2^ = 0.764, *F* = 60.613, *P* < 0.001). VIF values for all independent variables were below 5, indicating no severe multicollinearity. Residual diagnostics indicated acceptable normality and homoscedasticity; however, the Durbin–Watson value was 1.191, suggesting possible slight positive autocorrelation, which is acknowledged in the Limitations. Job role, number of published papers, attendance at an innovation leadership conference/training session, and critical thinking were independently associated with innovative leadership (*P* < 0.05). For brevity, Table 4 displays only variables with *P* < 0.05. Because of possible construct overlap between the ILS and CTDfn, common-method variance, and sample homogeneity, the model should be regarded as exploratory and requires validation in independent samples.

**Table 3:** Assignment table for categorical independent variables in multiple linear regression analysis.

Independent variables	Assignment
Average number of night shifts (per month)	≤1 (0,0,0,0); 2–4 (0,1,0,0); 5–7 (0,0,1,0); ≥8 (0,0,0,1)
Job role	Nurse (0,0,0); team leader (0,1,0); head nurse (0,0,1)
Employment status	Permanent staff (0,0,0); human resources services (0,1,0); contract employment (0,0,1)
Number of published papers	0 (0,0,0,0); 1–2 (0,1,0,0); 3–4 (0,0,1,0); ≥5 (0,0,0,1)
Attend an innovation leadership conference/training session	No = 1, Yes = 2
CTDfn scores	Substitute the original value

**Table 4:** Multiple linear regression analysis of factors associated with innovative leadership among male nurses (*N* = 222).

Variables	*B*	SE	*β*	*t*	*P*	95% CI		VIF
Constant	119.467	6.015	–	19.860	<0.001	107.608	131.325	–
Job role (team leader)	3.335	1.669	0.072	1.998	0.047	0.045	6.624	1.231
Job role (head nurse)	7.254	2.286	0.136	3.173	0.002	2.747	11.760	1.718
Number of published papers (1–2)	15.341	2.202	0.369	6.968	<0.001	11.000	19.681	2.626
Number of published papers (3–4)	22.254	3.499	0.376	6.360	<0.001	15.356	29.153	3.267
Number of published papers (≥5)	24.763	4.518	0.310	5.481	<0.001	15.856	33.671	2.994
Attend an innovation leadership conference/training session	6.426	2.626	0.166	2.447	0.015	1.248	11.603	4.328
CTDfn scores	0.305	0.074	0.178	4.118	<0.001	0.159	0.450	1.756

Dependent variable: innovative leadership total score. All candidate variables were entered using the Enter method. Only variables with *P* < 0.05 are displayed for brevity. Number of published papers was included as dummy variables with “0 papers” as the reference; job role with “nurse” as the reference; training attendance with “no” as the reference. All VIF values were <5, indicating no severe multicollinearity. Model summary: *R*^2^ = 0.777, adjusted *R*^2^ = 0.764, *F* = 60.613, *P* < 0.001, Durbin-Watson = 1.191.

## 4 Discussion

### 4.1 Male nurses' innovative leadership skills are at a moderate level

This study shows that male nurses' total score for innovative leadership was 154.00 (141.00, 168.00), with an average score of 3.58 (3.28, 3.91) across all items. Overall, the results were at a moderate level, slightly lower than those reported by Günay et al. (24). This difference may stem from various factors, including personal, environmental, and social factors. However, these possible explanations were not directly measured in the present study and should be regarded as hypotheses for future research rather than conclusions. At the individual level, some male nurses may lack systematic training in innovative leadership, face limited career advancement opportunities, and have low self-efficacy, all of which may hinder the development of innovative leadership skills. In the workplace, male nurses may be concentrated in high-pressure, high-intensity departments, where they devote most of their energy to completing routine tasks. Resources for general positions may be limited, and mechanisms for teamwork and communication may not yet be fully developed, which may hinder the implementation of innovative practices. At the sociocultural level, gender stereotypes and the low social recognition of the nursing profession may undermine male nurses' sense of professional belonging and motivation to innovate (25, 26). Therefore, nursing administrators should implement a comprehensive set of measures—including strengthening systematic training, optimizing the work environment, enhancing organizational support and incentive mechanisms, and improving public awareness to support the development of innovative leadership among male nurses.

### 4.2 Factors associated with innovative leadership among male nurses

The results of this study indicate that job role was associated with male nurses' innovative leadership. Team leaders and head nurses had significantly higher levels of innovative leadership than regular nurses, consistent with the findings of Fida et al. (27). They in managerial roles not only possess a higher sense of professional identity but also drive change and development in nursing practice by making decisions regarding departmental coordination, process optimization, and team management. Furthermore, they have an advantage in accessing resources, are more likely to be involved in hospital management decisions, and can promptly obtain cutting-edge industry innovation case studies and translate them into departmental practice (28). The number of published papers was also significantly associated with innovative leadership; male nurses who published more papers had higher levels of innovative leadership. Research activities may support the ability to apply professional knowledge and broaden innovative perspectives through the refinement of research approaches, the synthesis of evidence from the literature, and academic exchange, while also fostering the development of leadership and teamwork skills through interdisciplinary collaboration. Furthermore, research outputs such as published papers directly demonstrate innovative capabilities, thereby strengthening male nurses' credibility within their teams and the persuasiveness of their innovative practices (29). Male nurses who have participated in innovation leadership conferences or training programs demonstrate relatively higher levels of innovation leadership, consistent with the findings of Li et al. (30). The case studies and experience-sharing sessions during these events expose them to innovative practices in diverse contexts, broadening their horizons and sparking inspiration, which in turn helps them devise unique solutions (31). This suggests that nursing managers should tailor management strategies to the characteristics of different nursing groups to support the development of innovative leadership among male nurses.

### 4.3 Association between critical thinking and innovative leadership among male nurses

The results of this study indicate that critical thinking is positively correlated with innovative leadership (*P* < 0.01) and was independently associated with innovative leadership (*P* < 0.001). That is, the stronger the critical thinking skills of male nurses, the higher their level of innovative leadership. This may be because critical thinking enables nurses to identify problems with keen insight, avoid blindly accepting traditional nursing models, and conduct in-depth analysis and scrutiny of existing processes and methods, thereby uncovering opportunities for improvement and creating opportunities for innovation. At the same time, critical thinking helps nurses approach complex nursing issues from multiple perspectives, comprehensively consider various factors, propose scientific and effective innovative strategies, and rationally assess the feasibility and potential risks of innovative solutions to ensure the smooth implementation of innovative practices (32). Therefore, nursing managers should focus on cultivating critical thinking skills among male nurses. Through systematic training, case studies, and encouraging them to ask questions, they can provide opportunities for these nurses to exercise their minds, thereby supporting the development of innovative leadership and promoting the overall development of the nursing team.

### 4.4 Limitations and future research directions

This study has three main limitations. First, the cross-sectional design and convenience sampling mean that only associations at a single time point can be identified. Causal relationships cannot be established, and the findings are restricted to male nurses working in tertiary hospitals in Henan Province, China, rather than male nurses in general. Second, the exploratory model explained a high proportion of variance. This may be related to construct overlap between the ILS and CTDfn, common-method variance because all principal variables were self-reported in a single survey, conceptual proximity among predictors, and sample homogeneity due to convenience sampling. Therefore, the high *R*^2^ should be interpreted with caution and requires validation in independent samples. Third, although the primary analysis used enter multiple linear regression, stepwise regression was initially considered as an exploratory variable-selection strategy. Stepwise procedures can produce unstable models, inflate Type I error, yield biased coefficient estimates, and generate overly optimistic *R*^2^ values. Therefore, any stepwise-derived results should be regarded as exploratory only and require validation in independent samples.

For future research, stratified random sampling should be adopted to carry out multicenter, cross-regional investigations covering hospitals at all levels. Longitudinal tracking studies are recommended to clarify the causal sequence and dynamic change rules of male nurses' innovative leadership and its associated factors. Furthermore, follow-up research can add a control group of female nurses for comparative analysis, and adopt latent profile analysis to classify subgroups of male nurses based on their innovative leadership characteristics, so as to provide more precise, targeted references for nursing management interventions.

## 5 Conclusion

The innovative leadership of male nurses in tertiary hospitals of Henan Province remains at a moderate overall level. In this exploratory cross-sectional model, innovative leadership was associated with job role, academic output, relevant training experience, and critical thinking. To support the development of male nurses' innovative leadership capacity, nursing administrators should consider personalized strategies targeting these associated factors, develop systematic training focused on critical thinking and innovative practice, and support the diversified development of the nursing workforce and improvement of clinical nursing quality.

## Data Availability

The datasets used and/or analysed during the current study are available from the corresponding author on reasonable request.
